# Supplementary figures and images for: Effect of a hybrid team-based advanced cardiopulmonary life support simulation program for clinical nurses
Source: PLoS One. 2022 Dec 16;17(12):e0278512. doi: 10.1371/journal.pone.0278512 (PMC9757587; doi:10.1371/journal.pone.0278512)

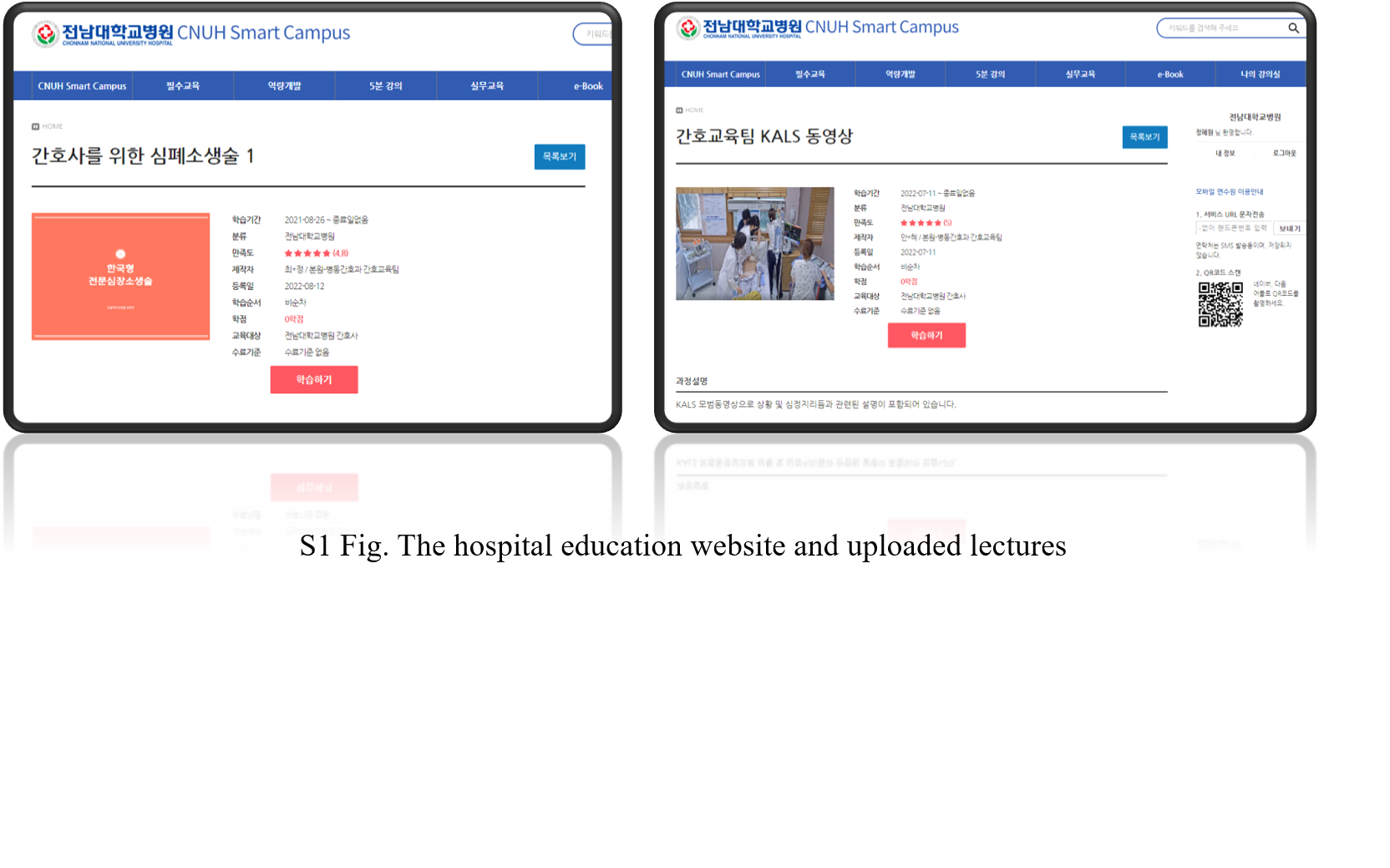

Supplement: S1 Fig — (TIF) [file pone.0278512.s001.tif]
