# Supplementary material for: Effect of a hybrid team-based advanced cardiopulmonary life support simulation program for clinical nurses
Source: PLoS One. 2022 Dec 16;17(12):e0278512. doi: 10.1371/journal.pone.0278512 (PMC9757587; doi:10.1371/journal.pone.0278512)
Supplement: Attachment — Submitted filename: Response to Reviewers_final.docx [file pone.0278512.s002.docx]

My co-authors and I sincerely appreciate the reviewers’ feedback. We have revised the manuscript to reflect the reviewers’ opinions as much as possible. The edits are highlighted in yellow in the manuscript.

| **Journal Requirements** | **Authors’ Response** | **Lines** |
| --- | --- | --- |
| The authors can add little more to support in development of the hypothesis. Make sure that hypothesis are framed in context of the population not to sample (participants). | Thank you for your kind review.  We have adapted our hypothesis to fit the population.  In the manuscript  This study’s hypotheses were that, compared with nurses undergoing a basic online ACLS program, nurses in the HTAS intervention group would have (1) superior ACLS performance (primary outcome) and (2) superior ACLS knowledge and CPR self-efficacy (secondary outcomes). | Revised manuscript  104-107 |
| In the result section, it is advised to add levene's test of homogeneity equality of variances in groups and test of normality as required for application to t test. | - We have added the normality test sentence to the Results section.  In the manuscript  Each group had more than 30 participants, the skewness of the outcomes was −0.49 to 0.38, and kurtosis was −1.70 to 2.12, so the analysis was performed assuming normality of the data.  - We have added Levene's test results from the t-test on independent samples.  In the manuscript  Levene’s test showed unequal variances (F = 9.99, p < 0.002), so the degrees of freedom were adjusted from 224 to 190.  Levene’s test indicated that the variances of the mean differences in ACLS knowledge, CPR self-efficacy, and CPR stress were equal (F = 0.98, p = 0.324; F = 0.20, p = 0.658; F = 0.01, p = 0.936, respectively). | Revised manuscript  283-285  292-293  312-314 |
| Please provide an amended statement that declares *all* the funding or sources of support | We have added a more explicit funding statement. |  |
| Please include your amended Funding Statement within your cover letter. | We have added a statement to the cover letter that there were no sources of funding other than the National Research Foundation of Korea (NRF) grant.  In the cover letter  The corresponding author received a National Research Foundation of Korea (NRF) grant funded by the Korea government for this study. There was no additional funding for this study. | Revised cover letter |
| Upload of research data | We uploaded the raw data to ‘protocols.io’. | https://protocols.io/file/jtf77qze.sav |
| Please include a separate caption for each figure in your manuscript | Figures have been included the manuscript. | Revised manuscript  121, 152, 190 |
| - | We found a coding error in the age row while checking the data, and Table 1 has been corrected accordingly. | Revised manuscript  287 |
| - | The author’s name of this study have been modified. | Revised manuscript  9-10 |
| **Reviewer #1 Comments** | | |
| **Comments** | **Authors’ Response** | **Lines** |
| **Abstract**  It should be detailed with all subtitles "Background, methods, results, conclusion" | We have added all of the appropriate headings to the abstract. | Revised manuscript  20-36 |
| **Abstract**  Add detailed data about the participants | This information has been added as requested.  In the manuscript  Of the 226 general ward nurses with more than 6 months of clinical experience, 117 were allocated to the intervention group (IG), which attended the HTAS, and 109 to the CG, which attended only basic ACLS training. | 27-36 |
| **Abstract**  The conclusion should be precise. Add the future directions. | The conclusion has been modified as requested.  In the manuscript  Conclusion: The HTAS for ward nurses was effective at enhancing the nurses’ ACLS performance. It is necessary to develop effective training methods for team-based ACLS and verify the sustained effects of such training. | 36-38 |
| **Introduction**  Define "a Hybrid Team-based CPR" in detail | We have defined "a Hybrid Team-based CPR" to the introduction.  In the manuscript  Hybrid CPR training includes a mixture of non-face-to-face methods using video or computer-based course material, along with face-to-face instructor-led training using CPR simulation [16]. A review of the effectiveness of alternative CPR training methods found hybrid methods to be more effective at facilitating knowledge retention and performance compared to standard instructor-led training[16]. Therefore, to improve the CPR competency of clinical nurses, an effective educational program might consist of a hybrid design that delivers ALCS knowledge and skills in an iterative and convenient way, allowing trainees to practice the acquired knowledge and skills on high-fidelity manikins. | 76-83 |
| **Introduction**  Explain the measured variables. | We have added the measured variables to the Introduction section.  In the manuscript  The outcomes of CPR training include the performance domain, consisting of elements such as chest compression rate and depth, hands-off time, and operation of defibrillators and bag-valve masks [5, 15]. The knowledge domain, on the other hand, comprises elements such as knowledge of the ACLS algorithm, electrocardiogram (ECG) analysis, and drug administration [5, 15, 17]. Finally, the attitudes domain consists of concepts such as self-efficacy and confidence [15]. A study of the effectiveness of team-based CPR simulation training found that such training enhanced satisfaction, attitudes, perceptions, and acquisition of knowledge and skills [9]. The effectiveness of team-based CPR simulation should be comprehensively evaluated based on important factors in each area of knowledge, skills, and attitudes. | 84-93 |
| **Introduction**  The significance of the study needs more details. | The Introduction section has been revised to clarify the purpose and importance of this study.  In the manuscript  Hybrid CPR training ~  In the manuscript  To increase the effectiveness of team-based CPR simulations, an online program that can conveniently and repeatedly deliver the ACLS guidelines can be combined with simulation training. A team-based non-face-to-face CPR education model rooted on KALS guidelines could provide ACLS education more conveniently and maintain its effectiveness. Thus, this study aimed to develop a hybrid team-based ACLS simulation (HTAS) training program mixed with online training content and to validate the effect of this training on nurses’ performance, knowledge, and self-efficacy. | 76-83  98-104 |
| **Introduction**  Add a clear hypothesis. | We have added the hypothesis to the Introduction section.  In the manuscript  This study’s hypotheses were that, compared with nurses undergoing a basic online ACLS program, nurses in the HTAS intervention group would have (1) superior ACLS performance (primary outcome) and (2) superior ACLS knowledge and CPR self-efficacy (secondary outcomes). | 104-107 |
| **Methods**  -The study design, ethics, and setting are not clear.  -Please, re-frame the components (SPICES) for methods | We have modified our overall research methods description.  The research methods description was revised to detail (1) the theory, (2) development of the program, and (3) verification of the effectiveness of the developed program.  In the third part belonging to the experimental research design, it was reframed with SPICES components. | Revised manuscript  overall |
| **Methods**  How and who administrates the data collection?  How did you achieve the validity and reliability of the outcome measures? | -In the subsection describing data collection, the part where the participant responded to the questionnaire and the part evaluated by the evaluator with the checklist have been separately described.  -It has been described in more detail to increase the reliability and validity of the outcome measures.  In the manuscript  The participants responded to the ACLS knowledge, CPR self-efficacy, and CPR stress online pre-questionnaire using a Google Forms survey. After the self-administered questionnaire was completed, the participants underwent the respective training programs designed for the IG and CG.  ACLS performance was assessed by evaluators from the research team. To establish concordance between two evaluators using five ACLS simulation videos, first, the two evaluators clearly agreed on the meaning of each item in the ACLS performance checklist. Second, after watching five ACLS simulation videos, two evaluators independently produced ACLS performance scores based on the checklist. The concordance between the scores of the two evaluators was a weighted kappa of 0.83. Third, when the two evaluators produced different performance scores after watching the ACLS simulation video, the reasons were discussed until a consensus was reached. After inter-rater agreement was reached, the ACLS performance was assessed. To evaluate the participants' ACLS performance, the evaluators assessed behavior with a checklist while watching the simulation in the same room with the participants.  After the ACLS performance assessment, the participants responded to the post-training questionnaire, which consisted of the same items as the pre-training questionnaire. | 211-227 |
| **Methods**  For statistical analysis, explain all methods used in detail and add the software used. | In the Methods section, the G*Power program used for calculating the sample size and the SPSS program used for data analysis have been mentioned.  In the manuscript  The sample size determination was based on the effect size used in a previous study that performed ACLS simulation education for clinical nurses [22]. The minimum number of participants needed to maintain the significance level (α = 0.05), effect size (f = 0.05), and power (1-ß = 0.95) was determined to be 210 using G*Power 3.1.9.4 software (Heinrich Heine University Düsseldorf, Düsseldorf, Germany). A 15% dropout rate was assumed, so the required sample size was increased to 241 participants: 121 participants in the intervention group (IG) and 120 participants in the comparison group (CG).  SPSS Statistics for Windows, version 26 (IBM Corp., Armonk, NY, USA) was used to analyze the study data. Descriptive statistics were used to analyze the participants’ demographic data, where means and standard deviations were calculated for continuous variables. The homogeneity of the participants’ general characteristics was verified using the χ2 test, Fisher’s exact test, or independent t-test analysis, as appropriate. The paired t-test and independent t-test were used to compare differences within and between outcomes. Given the differences in baseline stress, we also analyzed the data using analysis of covariance. The level of significance was set at p ≤ 0.05. | 163-169  272-279 |
| **Methods**  -What were the eligibility criteria for participants?  -Who enrolled participants? | The inclusion criteria for this study have been described.  In the manuscript  The participants were nurses who had worked in a general ward for more than 6 months and were recruited via convenience sampling. The reason for limiting the clinical experience of nurses to over 6 months was that, prior to this duration, nurses would not yet have adapted to their work routines [23], making it inappropriate to evaluate the educational effects of the HTAS in the presence of deficit baseline competency. | 172-176 |
| **Methods**  Mention the settings and locations where the data were collected | The hospital and ward where this study was conducted are described in detail, and the questionnaire response method and checklist measurement method are specified.  In the manuscript  During the study period, the hospital consisted of 19 medical and surgical wards, and a total of 17 wards participated, except for two designated as COVID-19 wards. All medical staff in this hospital were mandated to undergo CPR training once every 2 years, and if they participated in this study, it was recognized as one CPR training.  The participants responded to the ACLS knowledge, CPR self-efficacy, and CPR stress online pre-questionnaire using a Google Forms survey.  After inter-rater agreement was reached, the ACLS performance was assessed. To evaluate the participants' ACLS performance, the evaluators assessed behavior with a checklist while watching the simulation in the same room with the participants. After the ACLS performance assessment, the participants responded to the post-training questionnaire, which consisted of the same items as the pre-training questionnaire. | 159-162  211-212  222-227 |
| **Methods**  Provide sufficient details of interventions of each group to allow replication. | The HTAS development process and theory used in its development are described. | 125-153  194-202 |
| **Methods**  Define pre-specified primary and secondary outcome measures. | The outcomes were based on the hypotheses. The primary and secondary outcomes have been additionally described in relation to the hypotheses.  In the manuscript  This study’s hypotheses were that, compared with nurses undergoing a basic online ACLS program, nurses in the HTAS intervention group would have (1) superior ACLS performance (primary outcome) and (2) superior ACLS knowledge and CPR self-efficacy (secondary outcomes). | 104-107 |
| **Methods**  Explain with reasons for any changes to study outcomes after the study commenced. | There was no change in outcomes during the study. |  |
| **Methods**  How was the sample size determined? | The sample size calculation method has been described.  In the manuscript  The sample size determination was based on the effect size used in a previous study that performed ACLS simulation education for clinical nurses [22]. The minimum number of participants needed to maintain the significance level (α = 0.05), effect size (f = 0.05), and power (1-ß = 0.95) was determined to be 210 using G*Power 3.1.9.4 software (Heinrich Heine University Düsseldorf, Düsseldorf, Germany). A 15% dropout rate was assumed, so the required sample size was increased to 241 participants: 121 participants in the intervention group (IG) and 120 participants in the comparison group (CG). | 163-169 |
| **Methods**  -What was the method used to generate the random allocation sequence?  - Explain the type of randomization.  -Was there any restriction like blocking and block size?  - What kind of mechanism was used to implement the random allocation sequence?  - Were any steps taken to conceal the sequence?  -Who generated the random allocation sequence? | This study had a quasi-experimental design in which subjects were not randomly assigned. Therefore, we cannot describe the random allocation method technique. |  |
| **Methods**  -Who assigned participants? | Participants were assigned to the comparison group in the order in which they expressed their intention to participate by SMS.  In the manuscript  An educational notice was uploaded to the hospital website. According to the selection criteria, 239 eligible participants were recruited (Fig 3). For ACLS performance measurements, groups of five to six nurses were organized to form a total of 46 teams. To prevent treatment diffusion, participants were first assigned to the CG group in the order in which they expressed their intention to participate by SMS. After the CG's conventional education was completed, the IG's treatment was conducted. Ten participants in the CG and three participants in the IG were excluded due to incomplete answers. | 184-190 |
| **Methods**  - How was blinding addressed? | To prevent the treatment diffusion, the comparison group processes were conducted first, followed by those of the intervention group. | 186-188 |
| **Results**  - Results need to provide answers to the questions raised/researchable problem  - Results need to follow ABC (accuracy, brevity, clarity)  - Kindly frame it along with the following elements of results.  i. Text to tell the story  ii. Tables to summarize the evidence  iii. Figures to highlight the main findings | We describe our results based on the research questions. In the summary of the results, the tables are mainly used to present more information. We describe our results based on the research questions. In the summary of the results, the tables are mainly used to present more information. | Revised manuscript |
| **Results**  Kindly provide dates defining the periods of recruitment and follow-up. | Not applicable to this study. |  |
| **Results**  This section needs to be put in the line with objectives. | We have tried to describe the results according to the objectives. |  |
| **Results**  Explain Recruitment and Baseline data. | -The recruitment process is described in Methods.  -Baseline data are summarized in Table 1. | 172-190  287 |
| **Results**  Numbers analyzed need to be described well, especially in the column of "t and P values". | The statistical results are described in the tables 1–3. | 287, 300, 309 |
| **Results**  -Outcomes and estimation need to be explained well.  -Ancillary analyses and harms need to be addressed. | -We have tried to clearly state our results and estimates.  - Not applicable in this study. |  |
| **Discussion**  Introductory paragraph should include the main findings of the study. | Our main outcome was improved ACLS performance in the intervention group, which is described in the Discussion section.  In the manuscript  Various methods have been applied in attempts to improve CPR performance. In the context of simulation training, methods such as booster practice, feedback, and computer-controlled interactive manikins have been tested [30]. Other education strategies have included videos, popular songs, virtual reality content, gamification, and augmented reality [14, 15, 31]. Based on previous review studies, it can be said that there is sufficient evidence regarding the effectiveness of simulations, songs, and videos among various CPR education methods [14, 15, 30, 31]. Thus, our research team sought an effective and efficient education method to suit the current COVID-19 situation, and as a result, a hybrid method using online videos and simulations was adopted. Moreover, based on research on team-based simulation CPR education [9], our method focused on establishing theoretical frameworks, deliberate practices, and debriefing. | 329-339 |
| **Discussion**  This section needs to be put in the line with objectives and hypotheses. | The results of the hypothesis testing are described in the discussion.  In the manuscript  Various methods have been applied in attempts to improve CPR performance. ~ | 329-379 |
| **Discussion**  Explain the strengths and implications of the study in detail. | The strengths of this study are described in the discussion.  In the manuscript  Clinical nurses are often the first responders to IHCA events [4]; therefore, they must be trained to perform ACLS proficiently. As face-to-face education has become challenging due to the recent spread of COVID-19, in line with the changing times, here, a variety of CPR training materials—which traditionally would have focused on in-person simulation [6, 13]—was compiled to develop a non-face-to-face education program. Thus, the HTAS developed in this study is meaningful in that it is an attempt to ensure effective and varied CPR training for nurses through combining a variety of online content and field simulations. This study demonstrated that the HTAS improved nurses’ ACLS performance. | 321-328 |
| **Discussion**  The main limitation of the study design is not demonstrated. | The limitations of this study design are described.  In the manuscript  This study had some limitations. First, it was conducted on clinical nurses at general tertiary hospitals located in one region, and participants were selected through convenience sampling. Therefore, caution should be taken when generalizing the results of the study. The effect of such education could be confirmed more clearly in future studies if a randomized controlled trial approach with multi-institutional participation is adopted. Second, this study was a one-time education course conducted over a 1-week period; thus, the long-term effects of the program could not be measured. Further studies should verify the continuity of the effect of the HTAS intervention. Additionally, since reinforcement through repetition training is important in CPR training [12], the online content of this study can be utilized for repetitive training. | 397-406 |
| **Conclusion**  The conclusion should be precise. Add the future directions. | The future direction is that more diverse online learning content should be developed and tested to increase the efficiency of team-based simulations. This is described in the conclusion.  In the manuscript  In this study, a hybrid team-based ACLS education program, including online content, was developed for nurses. After undergoing training based on the developed program, the IG nurses’ ACLS performance improved compared to that of the CG, and when comparing the IG’s outcomes before and after the intervention, their ACLS knowledge and CPR self-efficacy showed improvements. Since the hybrid team-based ACLS training was effective at improving ACLS performance, future research on the efficiency of team-based simulation, incorporating more diverse non-face-to-face content, is warranted. If various online CPR educational materials are developed, it may be possible to maintain the quality of education and enable repeated and continuous education. | 409-417 |
| **Reviewer #2 Comments** | | |
| **Comments** | **Authors’ Response** | **Lines** |
| The title of the manuscript is novel and appropriately justified with methods and material. The findings and conclusions are properly matched with the objectives of the study. Some part of the manuscript needs to be rephrased as per the standard format. | Thank you for your kind review.  We have modified our overall research method. |  |
